# Novel vaccine strategies to induce respiratory mucosal immunity: advances and implications

**DOI:** 10.1002/mco2.70056

**Published:** 2025-01-16

**Authors:** Ming Zhou, Haiqin Xiao, Xinyi Yang, Tong Cheng, Lunzhi Yuan, Ningshao Xia

**Affiliations:** ^1^ State Key Laboratory of Vaccines for Infectious Diseases National Institute of Diagnostics and Vaccine Development in Infectious Diseases Xiang An Biomedicine Laboratory School of Life Sciences & School of Public Health Xiamen University Xiamen Fujian China

**Keywords:** multidimensional immune protection, respiratory mucosal vaccines, respiratory virus infection

## Abstract

Rapid advances in vaccine technology are becoming increasingly important in tackling global health crises caused by respiratory virus infections. While traditional vaccines, primarily administered by intramuscular injection, have proven effective, they often fail to provide the broad upper respiratory tract mucosal immunity, which is urgently needed for first‐line control of respiratory viral infections. Furthermore, traditional intramuscular vaccines may not adequately address the immune escape of emerging virus variants. In contrast, respiratory mucosal vaccines developed using the body's mucosal immune response mechanism can simultaneously establish both systemic and mucosal immunity. This dual action effectively allows the respiratory mucosal immune system to function as the first line of defense, preventing infections at the entry points. This review highlights the efficacy of respiratory mucosal vaccines, including innovative delivery methods such as nasal and oral formulations, in enhancing local and systemic immune barriers. Notably, respiratory mucosal vaccines offer potential advantages in protecting against emerging virus variants and maintaining long‐term and multidimensional immune memory in the upper respiratory tract. In addition, a combination of intramuscular and respiratory mucosal delivery of vaccines largely improves their coverage and effectiveness, providing valuable insights for future vaccine development and public inoculation strategies.

## Graphical abstract

An ideal respiratory virus vaccine should boost local and systemic protective effects and the persistence of multidimensional immunological memory. The respiratory mucosal vaccines may serve as an important supplement of traditional intramuscular vaccines

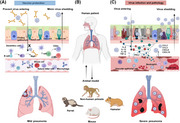

## 1 INTRODUCTION

Respiratory viral infections, whether pandemic or seasonal, pose a substantial global public health challenge, manifesting in varying degrees of severity. These infections range from viral replication in the upper and lower respiratory tracts to more severe outcomes, including pneumonia, multiple organ failure, and death.^1^ Historical outbreaks such as the 1918 influenza pandemic resulted in over 500 million infections and an estimated 50 to 100 million deaths.^2^ More recent outbreaks, including the 2002 SARS‐CoV epidemic, which caused 8,422 infections and 919 deaths,^3^ and the 2012 MERS‐CoV outbreak with 566 infections and 166 deaths, further illustrate the destructive potential of respiratory viruses.^4^ Since 2019, the SARS‐CoV‐2 pandemic has emerged as a critical global health crisis, with approximately 770 million confirmed cases and more than 7.05 million reported deaths to date. The enormous social and economic burden imposed by these respiratory viral infections emphasizes the urgent need for effective preventive strategies.

Although traditional intramuscular vaccines are effective in defending many infectious diseases, several challenges remain in the task of eliminating respiratory viral infection.^5^ These vaccines, which are typically administered via intramuscular or subcutaneous injections, primarily induce a systemic immune response, characterized by the production of circulating antibodies and activation of systemic immune cells. However, respiratory viral infections, such as influenza,^6^ respiratory syncytial virus (RSV),^7^ and SARS‐CoV‐2^8^ typically initiate infection in the upper respiratory tract mucosa. One of the key limitations of traditional intramuscular vaccines is their inadequacy in inducing robust respiratory mucosal immunity in the upper respiratory tract, which is the first line of defense against respiratory viruses. The mucosal immune system is distinct from the systemic immune system and relies heavily on the production of secretory immunoglobulin A (SIgA), localized immune cells, and mucosal barrier functions.^9^ Since traditional intramuscular vaccines are not administered at the respiratory mucosal surfaces, they often fail to effectively stimulate this crucial component of immunity. As a result, these vaccines may not prevent the initial stages of viral replication or transmission, even if they can protect against severe disease or systemic spread.^10^

In contrast, respiratory mucosal vaccines administered via the nasal or oral route can induce robust and long‐lasting protective respiratory mucosal immune responses that block virus entry and limit its spread in both the upper and lower respiratory tract.^11^ Moreover, respiratory mucosal vaccines are more accessible than traditional intramuscular vaccines, reducing needle‐related discomfort and simplifying the vaccination process, largely expanding the vaccine deployment efficiency and coverage, particularly in areas with high population density and scarce medical facilities.^12^

In light of these advantages, there has been growing interest in respiratory mucosal vaccine research over the past decade.^13^ This review aims to summarize and discuss recent advances in the development and application of respiratory mucosal vaccines, particularly for the SARS‐CoV‐2 pandemic, and to provide valuable insights for ongoing respiratory mucosal vaccine research against other respiratory viruses.

## 2 RESPIRATORY MUCOSAL STRUCTURE AND COMPONENTS

The respiratory mucosa is a complex tissue structure composed of epithelial cells, the basement membrane, the lamina propria, the submucosa, smooth muscle layers, and the adventitia.^14^ The epithelium, the outermost layer of the respiratory mucosa, contains ciliated columnar epithelial cells that help to clear mucus and trapped particles through coordinated wave‐like movements.^15^ Goblet cells in this layer secrete mucus that traps and removes inhaled pathogens and dust. In addition, microvilli on the surface of goblet cells are involved in detecting chemical signals and modulating local immune responses.^16^ Together, these epithelial cells form a protective barrier that prevents exogenous harmful substances, such as pathogens, from invading the host's respiratory tract.^17^ Impairment of the respiratory mucosal barrier usually results in increased susceptibility to viral infection by increasing the accessibility of viral receptors and facilitating more effective viral invasion.

**FIGURE 1:**
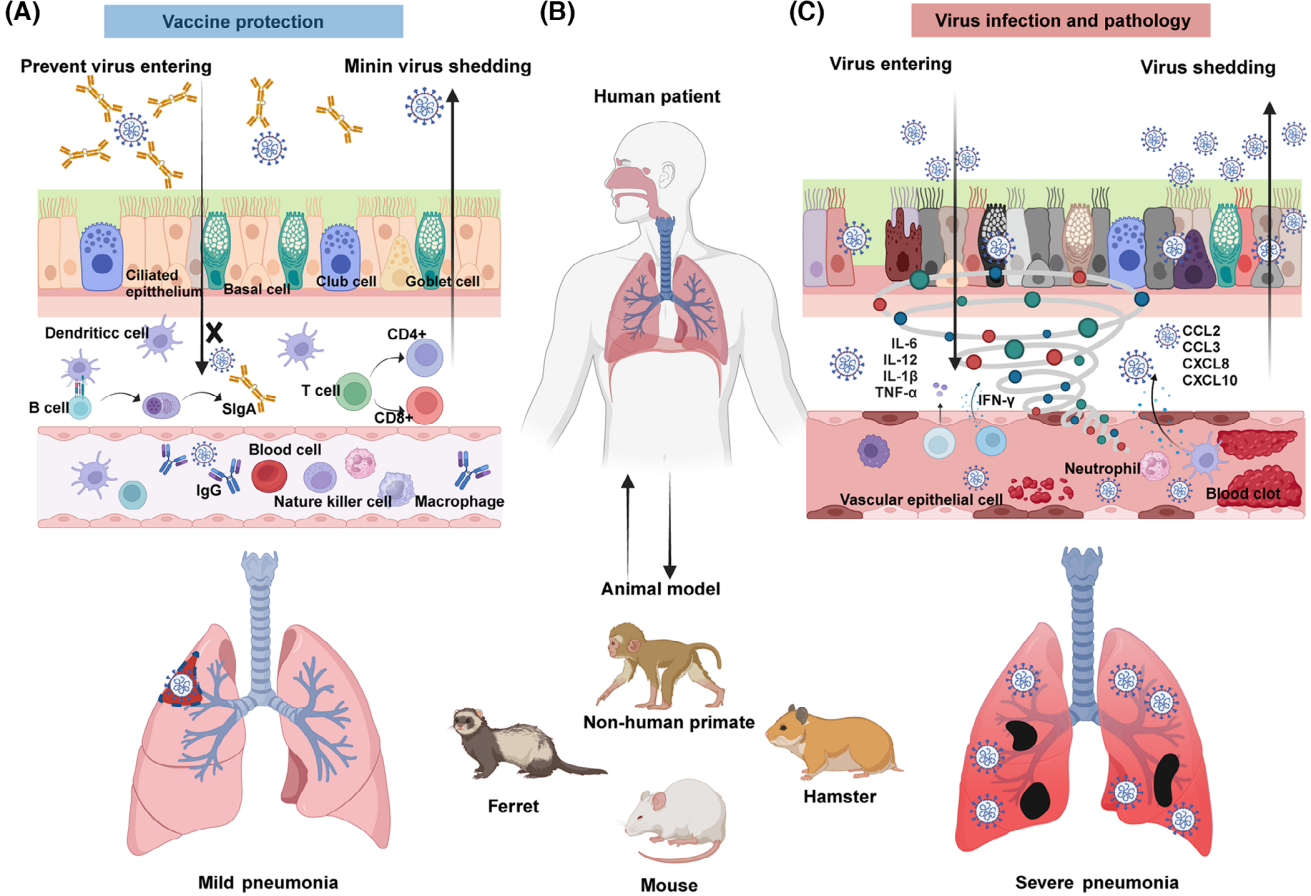
Schematic diagram of respiratory virus infection, pathology, and vaccine protection in human patient and animal model. (A) An ideal vaccine provides broad‐spectrum and long‐lasting protection against viral entry in the respiratory mucosal cells by SIgA, eliminating virus spread by circulating IgG, largely decreasing the severity of inflammation and tissue injury. (B) Animal models such as nonhuman primates, mice, ferrets, and Syrian hamsters are commonly used to study the diseases caused by respiratory virus infection and to evaluate vaccine efficacy. (C) Establishment of virus infection resulting in robust viral replication and shedding in the respiratory tract, cytokines storm, tissue injury, and death. Different colored cells represent the condition of cell damage: orange indicates normal cells, red indicates infected cells, and gray indicates cells that have died due to infection. Created with BioRender.com.

The respiratory mucosa also contains various immune cells, including macrophages, lymphocytes, and neutrophils, which are associated with the biological functions of immune defense and inflammatory responses.^18^ The establishment of virus infection in the respiratory mucosa usually affects the function, polarization state, number, and proportion of these immune cells. The mucosal injury that follows viral infection can amplify local inflammation, characterized by cell death, disruption of the immune response, release of cytokines, and chemotactic factors. Meanwhile, the virus may take advantage of this to increase its replication and spread (Figure 1).

## 3 IMMUNOLOGICAL BASIS OF RESPIRATORY MUCOSAL VACCINES

### 3.1 The role of innate immunity response in respiratory mucosal immunity

The mucosal barrier is the first line of defense in innate immunity. When the respiratory mucosal epithelium encounters a virus, mechanisms such as cilia and mucus play a critical role in filtering out pathogens. Respiratory mucus contains a number of antimicrobial substances that help to mitigate bacterial and viral infections.^14^ Once this first line of defense is breached, the innate immune system is rapidly activated, involving macrophages, neutrophils, and natural killer cells. Infected cells release numerous inflammatory mediators that trigger a broad immune response involving macrophages, T cells, and B cells to eliminate both the virus and damaged cells.^19^ At the same time, viral infection stimulates antiviral and inflammatory responses that upregulate interferon‐stimulated genes cytokines and chemokines associated with interferon signaling pathways and immune cell recruitment.^20^, ^21^ In addition, the complement system, another key component of the innate immune response, plays an important role in fighting viral infection and accelerating pathology.^22^ It works by directly lysing pathogens through a perforation mechanism and by enhancing the adhesion of macrophages and neutrophils to pathogen surfaces, thereby increasing the efficiency of phagocytosis and pathogen destruction.^23^ However, overactivation of the complement system often leads to thrombosis. In summary, the process of the innate immune response provides rapid and effective defense through mechanical, chemical, and cellular components, including physical barriers, antimicrobial peptides, enzymes, and cytokines. Various immune cells, such as macrophages, dendritic cells, mast cells, and natural killer cells, work together within this complex network to eliminate pathogens and maintain immune homeostasis.

### 3.2 The role of trained immunity against respiratory virus infection

Beyond direct antiviral effects, trained immunity is a crucial aspect of the innate immune response's defense mechanisms. Trained immunity refers to a form of innate immune memory in which initial activation of the innate immune system results in an enhanced response to subsequent antigenic challenges.^24^ This concept is particularly relevant to respiratory mucosal immunity, where trained immunity factors are closely related to the severity of disease caused by respiratory viral infections^25^ The respiratory mucosal tissue is abundant in mucus‐secreting glands and various innate immune cells, including natural killer cells, innate lymphoid cells, monocytes, macrophages, and dendritic cells.^26^ Once immune memory is established, these cells can mount a nonspecific immune response to subsequent exposures to the same or different antigens. Exposure to viral antigens and vaccination are the primary methods of inducing trained immunity in the respiratory mucosa.^27^ For example, COVID‐19 convalescent patients exhibit a memory phenotype in CD16^+^ and CD14^+^ monocytes, characterized by increased chromatin accessibility for IL1β, IL6, and IL8.^28^ Individuals with breakthrough SARS‐CoV‐2 infections have higher levels of IgA^+^ memory B cells in the upper respiratory tract, suggesting a localized and potentially more effective immune response at respiratory mucosal surfaces.^29^ Acute respiratory viral infections can lead to the formation of long‐lived memory alveolar macrophages that are reprogrammed to express high levels of MHC class II and produce elevated levels of neutrophil chemokines upon subsequent antigenic stimulation, thereby enhancing antiviral responses.^30^ Trained immunity‐induced reprogramming and epigenetic modifications may provide cross‐protection against different pathogens. For example, the bacillus Calmette‐Guérin vaccine has been reported to provide some protection against COVID‐19,^31^ and influenza vaccines have shown cross‐protection against SARS‐CoV‐2 infection.^32^

### 3.3 The role of immunoglobulin A in respiratory mucosal immunity

Immunoglobulin A (IgA) is one of the major antibody components of the mucosal immune system and is the most abundantly produced antibody molecule.^33^ Synthesized by plasma cells, IgA molecules traverse respiratory mucosal epithelial cells via transcytosis to reach the respiratory mucosal surface, where they are converted into SIgA.^34^ SIgA is the predominant immunoglobulin on respiratory mucosal surfaces in mammals, which performs several critical functions^35^: (1) It competes with foreign antigens for binding sites on epithelial surfaces^36^; (2) thereby inhibiting viral adhesion; (3) SIgA forms complexes with antigens within the lamina propria of the mucosa, which are then secreted to the respiratory mucosal surface and cleared by mucociliary action, effectively neutralizing foreign antigens^37^; (4) SIgA can directly reduce inflammatory responses by inhibiting the effector functions of inflammatory cells, thereby providing protection against viral infection.^38^ The primary advantage of respiratory mucosal vaccine‐induced SIgA is its ability to block direct contact between viruses and the respiratory mucosa, thereby preventing systemic viral infections. Traditional intramuscular vaccination strategies often struggle to induce SIgA in the respiratory mucosa.^39^ However, vaccines that specifically target the respiratory mucosa may directly stimulate the production of pathogen‐specific SIgA, particularly in the upper respiratory tract.^40^ Respiratory mucosal inoculation routes, such as nasal spray and inhalation, effectively deliver the vaccine components to both upper and lower respiratory tracts, inducing both respiratory mucosal SIgA and systemic IgA, thus providing multiple protection against pathogen evading.^41^

### 3.4 The role of cellular immune response in respiratory mucosal immunity

T cell‐mediated cellular immunity involves antigen‐specific responses in which T cells differentiate, proliferate, and transform into effector T cells upon encountering antigens.^42^ These effector T cells exert direct cytotoxic effects and coordinate synergistic killing activities against antigens^43^ Based on their spatial characteristics, functional T cells are generally categorized into circulating T cells and tissue‐resident memory T cells (TRMs).^44^ Mature circulating T cells circulate through the circulation system to peripheral immune organs and can recirculate through lymphatic vessels, peripheral blood and interstitial fluids to perform cellular immune and regulatory functions.^45^ In contrast, TRM cells are localized in different tissues and organs and mediate localized immune responses against invading pathogens. When neutralizing antibody production is insufficient to counteract viral infections, TRMs in the respiratory tract and lungs become crucial for controlling respiratory viral infections. A recent study showed that TRMs provide more immediate and robust protective immunity compared with circulating T cells.^46^ Respiratory mucosal vaccines are effective in inducing TRM recruitment in respiratory tract organ tissues.^47^ As the upper respiratory tract is the initial site of contact and infection with respiratory viruses, TRMs residing in the nasal epithelium may inhibit the progression of the virus from the upper respiratory tract to the lungs, thereby preventing the progression of pulmonary and systemic disease.^48^ Therefore, the recruitment and location of TRMs are integral to the antiviral and immune regulation functions of respiratory mucosal vaccines.

### 3.5 Mucosal immunity serves as an important complement to systemic humoral immunity

**FIGURE 2:**
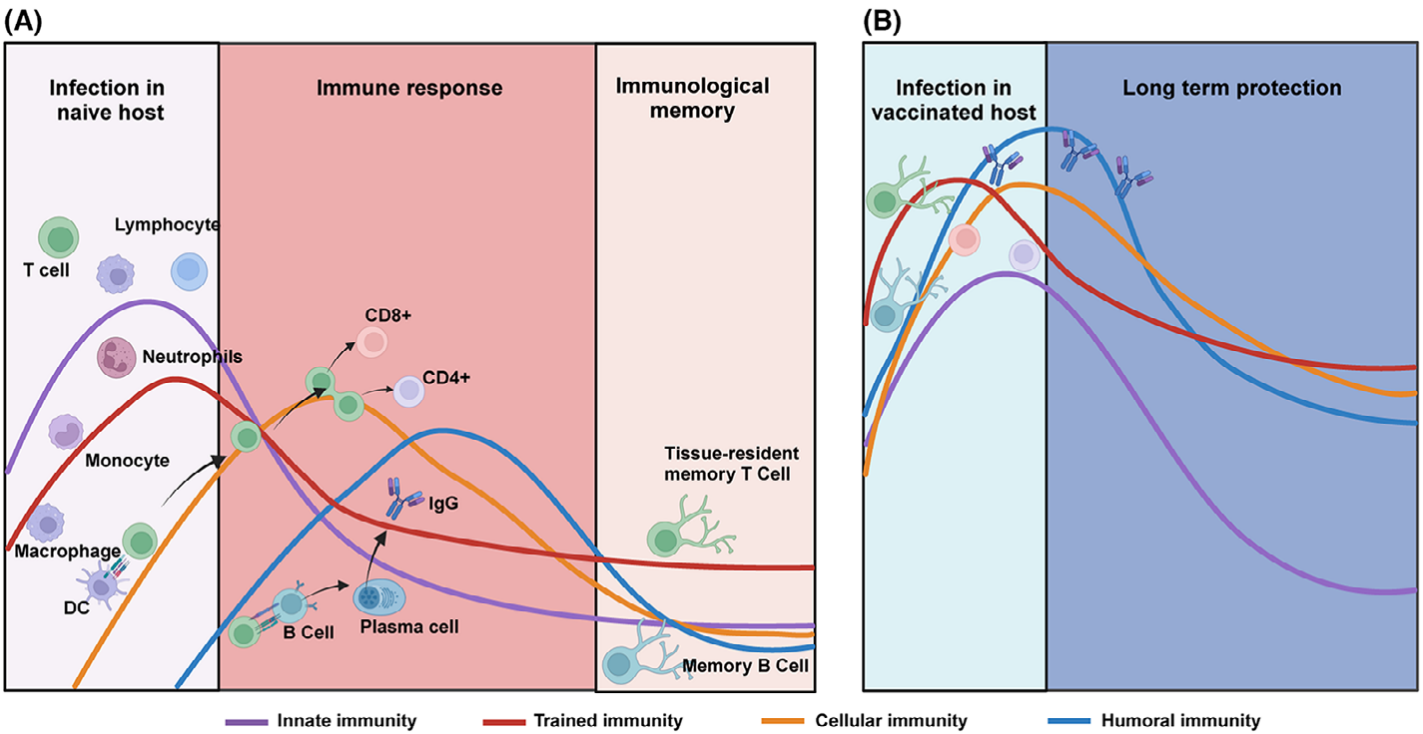
Schematic representation of different immune responses to respiratory virus infection in the naive and vaccinated hosts. (A) In the naive host, respiratory virus infection triggers rapid activation of innate immune responses in the epithelial cells and immune cells including macrophages, monocytes, dendritic cells, and neutrophils. In addition, exposure to pathogens usually induces the memory of nonspecific innate immunity, that is, trained immunity. Subsequently, the presentation of viral antigens induces adaptive immune responses. Activation of cellular immunity is characterized by the recruitment and activation of CD8^+^ cytotoxic T cells and CD4^+^ helper T cells. In addition, B cells differentiate into mature plasma cells that produce virus‐specific antibodies such as IgG and SIgA, which are potent in neutralizing virus particles and preventing virus spread. (B) Vaccination may boost a stronger and longer‐lasting immune response than viral infection, which is an option for establishing herd immunity against certain respiratory viruses. Created with BioRender.com.

Humoral immunity, which involves the production of antibodies by plasma B cells, plays a crucial role in systemic immune protection against viral infections.^49^ This form of host immune response is the key determinant of the protection efficacy of the traditional intramuscular vaccines, which rely primarily on B cells to generate pathogen‐specific IgG. However, due to anatomical limitations, IgG induced by systemic humoral immunity can rarely reach the mucosal regions of the respiratory tract, limiting their ability to neutralize viruses. Moreover, the constant mutation of the virus can lead to evasion of IgG, resulting in a continuous reduction in the protective effect of humoral immunity.^50^, ^51^ Humoral responses also have a slower onset compared with innate or cellular immunity, which limits their ability to counteract viral infections during the early stages of exposure. Given these limitations, there is an urgent need to develop novel vaccination strategies to improve protection effectiveness against emerging variants with increasing immune escape capacity. Therefore, respiratory mucosal vaccines that act directly in mucosal regions serve as a critical complement to the systemic immune response by inducing localized immune responses at respiratory mucosal surfaces, effectively bridging the gap left by traditional intramuscular vaccines and providing enhanced protection against viruses that infect respiratory mucosal tissues (Figure 2).

**FIGURE 3:**
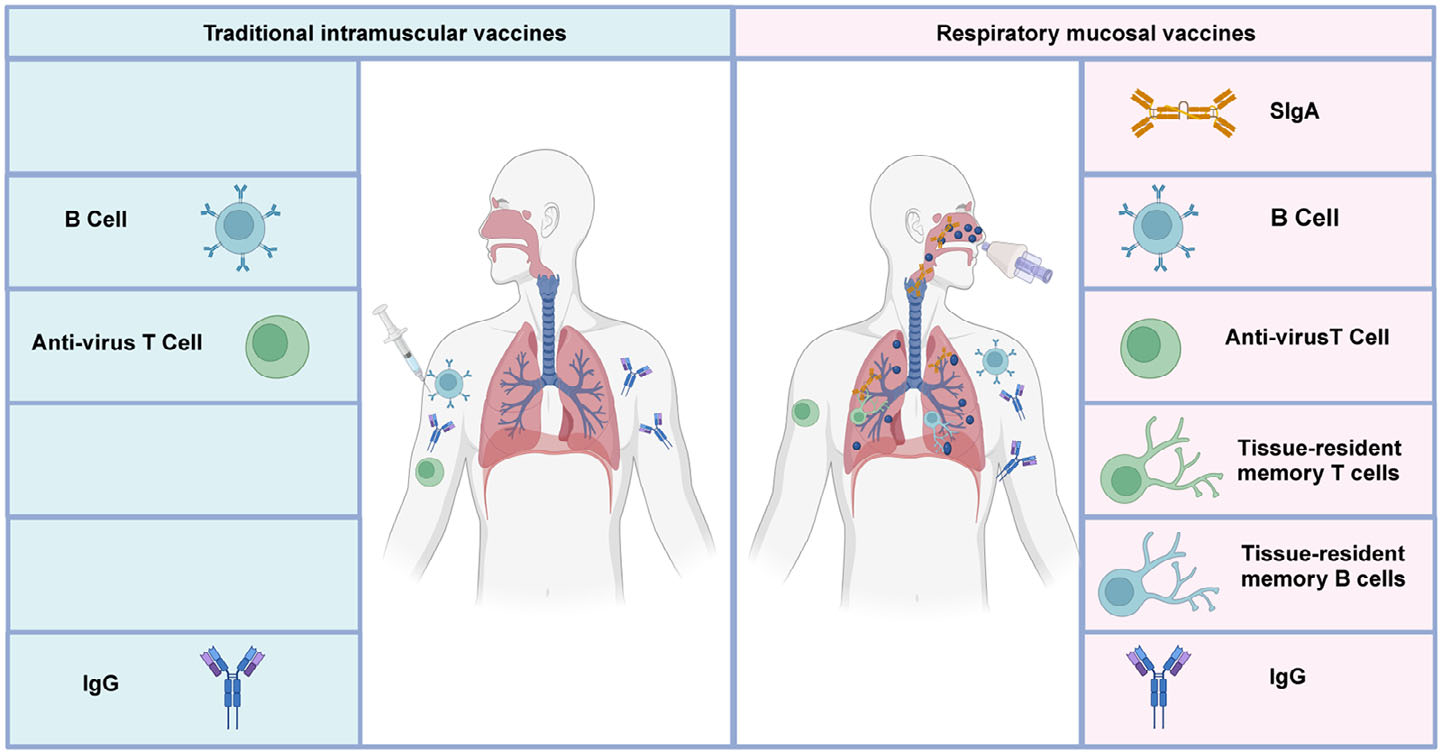
Differences in immune response induced by traditional and respiratory mucosal vaccines. Traditional intramuscular vaccines, typically administered via injection, predominantly elicit systemic immune responses, characterized by circulating B cells, antivirus T cells, and IgG antibodies, which do not significantly induce mucosal immunity in the upper respiratory tract. Respiratory mucosal vaccines, delivered through mucosal surfaces (e.g., nasal spray), are shown to elicit both systemic and mucosal immune responses. In addition to B cells and antivirus T cells, mucosal vaccines trigger the production of secretory IgA and tissue‐resident memory cells, including memory T cells and memory B cells, which reside in the mucosal tissues and offer localized protection at the site of viral entry, particularly in the upper respiratory tract. Created with BioRender.com.

Respiratory mucosal vaccines stimulate both innate and adaptive immune responses. The innate immune response is activated immediately upon vaccine administration, triggering dendritic cells and macrophages to present antigens in mucosal‐associated lymphoid tissues.^52^ This sets the stage for the adaptive immune response, which involves the production of SIgA and the activation of cytotoxic T lymphocytes (CTLs).^53^, ^54^ The SIgA remain localized at respiratory mucosal surfaces and prevent viral adherence and invasion. Meanwhile, CTLs actively seek out and destroy infected cells, eliminating viral reservoirs. Vaccination strategies that stimulate this type of immunity have the potential to transform the control of respiratory infections, reduce transmission, and offer durable protection.^55^ Mucosal immunity is especially relevant in improving vaccine design against respiratory viruses, addressing the limitations of traditional intramuscular vaccines that primarily induce systemic immunity (Figure 3).

## 4 ADVANCES IN RESPIRATORY MUCOSAL VACCINES STRATEGIES

The extensive coverage of the respiratory tract mucosal surfaces provides multiple routes of vaccination. Currently, respiratory mucosal vaccines are usually inoculated by the routes of intranasal transfusion, nasal inhalation, oral ingestion, and others.^16^ These routes of administration can elicit different patterns of protective immune responses, highlighting the need for further research to optimize respiratory mucosal vaccine strategies.

### 4.1 Intranasal transfuse inoculation

Intranasal transfuse inoculation of respiratory mucosal vaccines involves directly administering a vaccine containing the immunogen, either alone or combined with adjuvants into the nasal cavity in liquid form. Critical information including the route of vaccine immunization; animal model used in research; vaccine dosage and immune schedule, et al of recent studies in respiratory mucosal vaccines administered via intranasal transfuse are summarized (Table 1). For instance, Zhang et al.^56^ conducted nasal vaccinations using a chimeric SARS‐CoV‐2 receptor binding domain (RBD) nanoparticle vaccine in K18‐hACE2 transgenic mice and rhesus macaques, observing a robust respiratory mucosal immune response and cross‐protection against various SARS‐CoV‐2 variants in both models. Fu et al.^57^ employed a self‐healing hydrogel vaccine containing SARS‐CoV‐2 RBD. The highly bioadhesive hydrogel vaccine increases antigen stability and prolongs residence time in the nasal cavity and lungs by confining the antigen to the surface of the nasal mucosa, thereby enhancing antigen‐specific humoral, cellular, and mucosal immune responses. Boley et al.^58^ developed a subunit respiratory mucosal vaccine containing SARS‐CoV‐2 RBD and nucleocapsid (N) protein or their mRNA along with sodium urate adjuvant. This vaccine led to the rapid clearance of respiratory viruses and significantly reduced levels of proinflammatory cytokines in the lung tissue of vaccinated elderly ferrets, demonstrating effective protection. Hartwell et al.^59^ developed an innovative protein immunogen vaccine using amphiphilic albumin‐conjugated polymer‐lipid tails (Amph‐proteins). The SARS‐CoV‐2 RBD was conjugated to the Amph carrier to form the Amph‐RBD vaccine. Intranasal transfuse inoculation in mice and macaques resulted in neutralizing antibodies in serum, nasal lavage, and bronchoalveolar lavage fluid, and elicited antigen‐specific IgG and IgA responses in macaques. Zheng et al.^60^ developed a biomimetic viral nanoparticle vaccine for nasal administration aimed at inducing mucosal immunity against respiratory viruses. This vaccine includes poly(I:C) as an adjuvant, mimicking viral genetic material, and biomimetic pulmonary surfactant (bio‐PS) liposomes as the viral coat, along with SARS‐CoV‐2 RBD. Nebulization simulates the SARS‐CoV‐2 infection process. Following vaccination in C57BL/6 mice, a five‐fold increase of secretory SIgA was detected in respiratory secretions, presenting a novel method for preventing respiratory virus infections. Ke et al.^61^ used a highly attenuated vesicular stomatitis virus vector encoding the SARS‐CoV‐2 spike protein (VSVMT‐S). A single intranasal dose of the VSVMT‐S vaccine in Syrian hamsters elicited potent SARS‐CoV‐2‐specific neutralizing antibodies. Van et al.^62^ developed an intranasal SARS‐CoV‐2 subunit vaccine featuring a recombinant, proline‐stabilized D614G spike protein (mC‐Spike) linked to outer membrane vesicles (OMVs) derived from Neisseria meningitidis. Intranasal inoculation of BALB/c mice and Syrian hamsters with the OMV‐mC‐Spike vaccine induced a six‐fold increase of IgG and IgA in serum, nasal lavage, and lung tissues. An et al.^63^ reported a nasal subunit vaccine utilizing lyophilized SARS‐CoV‐2 spike protein and liposome‐stimulating adjuvants (nano‐STING‐spike protein). A single intranasal dose in mice model induced systemic neutralizing antibodies and specific T cell responses in lung and nasal lavage. Stauft et al.^64^ designed an attenuated nasal vaccine using a SARS‐CoV‐2 vector with three genomic modifications: removal of the furin cleavage site, open reading frame 6–8 knockout, and mutations in the nonstructural proteins 1 gene. This vaccine induced local mucosal and systemic IgA and IgG in Syrian hamsters and is currently undergoing Phase I clinical trials to verify its protective efficacy in humans.

**TABLE 1:** Advances in intranasal transfuse inoculation vaccine strategies and clinical information.

Route of immunization	Animal used in research	Vaccine	Adjuvants	Dosage and immune schedule	Immune response and testing sample	Phase	Ref
Intranasal transfuse inoculation	K18‐hACE2 mice and rhesus monkey	Helicobacter pylori ferritin with SARS‐CoV‐2 RBD	‐	Two dose immune in D0, D28	Systemic response and mucosal response in serum, BALF, and splenocytes	Preclinical	^56^
	BALB/c	RBD adjuvanted with aluminum hydroxide	Aluminum ions	Two dose immune in D0, D21	Systemic response and mucosal response in serum, BALF, and splenocytes	Preclinical	^57^
	Ferrets	Lipid nanoparticle‐based RBD–mRNA	Monosodium urate	Two dose immune in D0, D21	Mucosal response in serum, BALF	Preclinical	^58^
	BALB/c mice	Amphiphilic albumin‐conjugated polymer‐lipid tails‐RBD	‐	Two dose immune in D0, D28	Systemic response and mucosal response in serum, BALF, and splenocytes	Preclinical	^59^
	C57BL/6	Bionic‐virus with SARS‐CoV‐2 RBD	Poly(I:C)	Three dose immune in D0, D3, D15	Mucosal response in serum or BALF	Preclinical	^60^
	hACE2‐mice and Syrian hamsters	Vesicular stomatitis virus vector encoding the SARS‐CoV‐2 spike	‐	Single‐dosed	Systemic response and mucosal response in serum, BALF, and splenocytes	Preclinical	^61^
	BALB/c and Syrian hamsters	Spike protein linked to outer membrane vesicles	‐	Two dose immune in D0, D21	Mucosal response in serum, nasal washes, and lung homogenates	Preclinical	^62^
	BALB/c mice	SARS‐CoV‐2 spike protein and liposome‐stimulating adjuvants	STING	Single‐dosed	Mucosal response in serum, nasal washes, lung homogenates, and splenocytes	Preclinical	^63^
	Syrian hamsters	Attenuated SARS‐CoV‐2	‐	Single‐dosed	Systemic response and mucosal response in serum, BALF and splenocytes	Phase 1	^64^
Intranasal transfuse inoculation	BALB/c and K18‐hACE2 mice	Adenovirus vectors AD5 and AD19a, encoding SARS‐CoV‐2 S or N proteins	‐	Single‐dosed	Mucosal response in serum, BALF and lung homogenates and splenocytes	Phase 3	^78^, ^79^
	Syrian hamsters	SARS‐CoV‐2‐mRNA‐LNP	‐	Two dose immune in D0, D21	Mucosal response in serum	Preclinical	^80^
	Syrian hamsters	replication‐deficient VSV vector encoding SARS‐CoV‐2 spike	‐	Two dose immune in D0, D21	Systemic and mucosal response in serum, nasal washes, lung homogenates and splenocytes	Preclinical	^81^
	BALB/c mice	Triple‐RBD scaffold protein with flagellin	Flagellin protein	Two dose immune in D0, D21	Systemic and mucosal response in serum, nasal washes, lung homogenates and splenocytes	Preclinical	^88^
	BALB/c and hACE2‐mice	Ferritin nanoparticle vaccine	‐	Three dose immune in D0, D21, D42	Systemic response and mucosal response in serum and splenocytes	Preclinical	^95^
	Syrian hamsters	SARS‐CoV‐2‐LNP	‐	Two dose immune in D0, D21	Systemic response and mucosal response in serum and splenocytes	Preclinical	^97^
	BALB/c mice	Chimpanzee adenovirus vector expressing harbouring DNA encoding spike	‐	Three dose immune in D0, D21, D28	Systemic response and mucosal response in serum, BALF and splenocytes	Preclinical	^100^

Abbreviations: BALF, bronchoalveolar lavage fluid, RBD, receptor binding domain; VSV, Vesicular stomatitis virus.

### 4.2 Nasal inhalation inoculation

Nasal inhalation inoculation is a technique that converts liquid vaccines into fine aerosol particles that are then inhaled to elicit a more stable, robust, and longer‐lasting immune memory. This method requires specialized nebulizers to generate micro‐sized aerosol droplets from the liquid vaccine. Once inhaled, these droplets target the respiratory tract, where they induce immune responses within the respiratory mucosal lining and impede virus invasion. Critical information including the route of vaccine immunization, animal model used in research, vaccine dosage, and immune schedule, etc., of recent studies in respiratory mucosal vaccines administered via nasal inhalation are summarized (Table 2). For example, Xu et al.^65^ developed a replication‐deficient adenovirus‐based recombinant coronavirus vaccine (Ad5‐nCoV). Ad5‐nCoV was delivered in prefilled syringes and elicited strong systemic and mucosal immune responses when administered by nasal spray to rhesus macaques. Ad5‐nCoV has shown excellent tolerability and safety in Phase III clinical trials.^66^ Zhu et al.^67^ generated a nasal spray SARS‐CoV‐2 vaccine derived from an NS1 gene deleted live influenza virus vector carrying the SARS‐CoV‐2 RBD (dNS1‐RBD), which was inoculated using a special sprayer that atomized the liquid into a fine mist of droplets 10–70 µm in diameter. Afterward, Deng et al.^68^ confirmed that the dNS1‐RBD vaccine provides broad‐spectrum protection against different SARS‐CoV‐2 variants in mouse and hamster models. The dNS1‐RBD vaccine demonstrated substantial protective effects in mouse and hamster models by establishing innate immunity, trained immunity, and tissue‐resident memory T cells in the upper and lower respiratory tract through multiple immune response mechanisms.^69^ Prior to its approval, serial clinical trials confirmed its favorable safety and efficacy outcomes. Sunagar et al.^70^ developed a nasal SARS‐CoV‐2 vaccine using a chimpanzee adenovirus vector (ChAdOx1‐S). Preclinical studies in mice, rats, Syrian hamsters, and rabbits indicate strong mucosal and systemic humoral and cellular immune responses, and phase III clinical trials confirm its safety profile.^71^ Tioni et al.^41^ reported a gene‐engineered nasal spray vaccine using an attenuated respiratory syncytial virus vector to deliver the SARS‐CoV‐2 spike protein. Immunization of African green monkeys resulted in potent neutralizing antibodies against homologous viruses and cross‐neutralization against variants. This vaccine is currently in Phase I clinical trials. Le Nouen et al.^72^ developed a live attenuated vaccine using a parainfluenza virus vector expressing the SARS‐CoV‐2 spike protein (B/HPIV3/S‐6P). Evaluation in rhesus macaques showed that a single dose of B/HPIV3/S‐6P induced specific IgA and IgG responses in the respiratory mucosa, along with robust specific antibodies in the serum and strong systemic and lung‐specific CD4^+^ and CD8^+^ T cell responses.

**TABLE 2:** Advances in nasal inhalation inoculation, other respiratory mucosal vaccine strategies, and clinical information.

Route of immunization	Animals used in research	Vaccine	Adjuvants	Dosage and immune schedule	Immune response and testing sample	Phase	Ref
Nasal inhalation inoculation	Rhesus macaques	Adenoviral vector vaccine encoding the SARS‐CoV‐2 spike	TLR7/8	Three dose immune in D0, D14, D28	Systemic response and mucosal response in serum, BALF	Phase 3	^65^, ^66^
	BALB/c and Syrian hamsters	Replication deficient influenza a virus expressing RBD domain of spike protein	‐	Two dose immune in D0, D28	Systemic response and mucosal response in serum, BALF and splenocytes	Emergency Use Listing	^67^, ^68^
	BALB/c and K18‐hACE2 mice	Adenoviral vector expressing harboring DNA encoding spike	‐	Single‐dosed	Systemic response and mucosal response in serum, BALF and splenocytes	Phase 3	^70^, ^71^
	African green monkeys and K18‐hACE2 mice	Attenuated respiratory syncytial virus vector to deliver the SARS‐CoV‐2 spike protein	‐	Single‐dosed	Systemic response and mucosal response in serum, BALF and splenocytes	Phase 1	^41^
	rhesus macaques	Live attenuated vaccine using a parainfluenza virus vector expressing the SARS‐CoV‐2 spike	‐	Single‐dosed	Systemic response and mucosal response in serum, BALF and splenocytes	Phase 1	^72^
	BALB/c and Syrian hamsters and cynomolgus monkey	CTB self‐assembled nanoparticles bearing the SARS‐CoV‐2 RBD	‐	Two dose immune in D0, D14	Systemic response and mucosal response in serum, BALF	Preclinical	^87^
Other mucosal inoculation routes	BALB/c and K18‐hACE2 mice	Dissolving microneedles to administer the SARS‐CoV‐2 S	‐	Single‐dosed	Mucosal response in serum, BALF	Preclinical	^73^
	Syrian hamsters	Replication‐deficient Adenovirus vector expressing SARS‐CoV‐2 antigens	‐	Two dose immune in D0, D28	Systemic response in serum	Phase 2	^74^

Abbreviation: CTB, Cholera toxin B.

Intranasal transfuse and nasal inhalation are the commonly used methods for respiratory mucosal vaccine administration. Intranasal transfuse inoculation, as a straightforward delivery method of respiratory mucosal vaccines, offers notable advantages including simplicity and convenience.^73^ Besides intranasal transfuse and nasal inhalation, other respiratory mucosal inoculation routes have been developed to effectively induce respiratory mucosal immunity, aiming to strengthen immune responses at the respiratory mucosal surfaces for better protection against respiratory infections.^74^ Oral immunization as one of the earliest methods of vaccine administration, could date back to the late 19th and early 20th centuries.^75^ The long history of oral vaccine development highlights its advantages, including ease of administration, cost‐effectiveness, and the potential to stimulate mucosal immunity. However, intranasal transfuse inoculation also presents challenges. Its effectiveness may vary due to individual differences in nasal mucosal conditions and anatomical structures. To achieve an effective intranasal immunization, the liquid of vaccine components should be adequately absorbed by the mucous membranes. Incorrect administration or anatomical anomalies may result in reduced absorption efficiency and suboptimal immune responses. Therefore, while intranasal transfuse inoculation has shown promise in preclinical research, these barriers in the translation process need to be addressed to facilitate its successful implementation in clinical practice.

### 4.3 Other respiratory mucosal inoculation routes

In addition to respiratory inoculation, respiratory mucosal vaccines can be administered by other routes, including oral, sublingual, and ocular applications (Table 2). Kim et al.^76^ utilized dissolving microneedles to administer the SARS‐CoV‐2 S1 protein subunit to the sublingual area. This approach induced robust mucosal immune responses in K18‐hACE2 mice, alleviated pulmonary inflammation, and mitigated the cytokine storm caused by SARS‐CoV‐2 infection. Susan et al. developed an oral vaccine based on a replication‐deficient adenovirus vector expressing SARS‐CoV‐2 antigens, combined with a novel Toll‐like receptor 3 agonist as an adjuvant. Oral gavage of this vaccine in hamsters resulted in specific IgG antibody responses and protection against SARS‐CoV‐2 infection.

### 4.4 Integrated inoculation strategy of respiratory mucosal and intramuscular vaccinations

Recently, mucosal vaccines have been increasingly used as booster doses to increase the protective efficacy of conventional intramuscular vaccines. Dennis et al. reported the use of adenovirus vectors AD5 and AD19a, encoding SARS‐CoV‐2 S or N proteins, as boosters following primary intramuscular immunization with heterologous DNA or mRNA vaccines. This approach effectively stimulated both systemic and mucosal immune responses in mice, with nasal boosting resulting in elevated levels of mucosal IgA and pulmonary TRM cells.^75^, ^76^ Mao et al.^77^ developed a strategy to enhance mucosal memory by administering an unadjuvanted nasal vaccine to individuals with pre‐existing immunity from primary intramuscular vaccinations. Their study demonstrated that K18‐hACE2 mice initially immunized with a muscle‐administered mRNA‐lipid nanoparticle (LNP) vaccine, exhibited potent induction of resident memory B and T cells and mucosal IgA following a subsequent unadjuvanted SARS‐CoV‐2 spike protein nasal vaccination. Zhang et al.^78^ investigated the efficacy of heterologous boosting using a respiratory mucosal vaccine with a replication‐deficient VSV vector encoding SARS‐CoV‐2 spike protein following two doses of intramuscular inoculation of inactivated SARS‐CoV‐2 vaccine. Results showed that heterologous boosting by the intranasal route significantly enhanced both systemic and mucosal immune responses in hamsters.

## 5 ADJUVANTS AND VECTORS ENHANCE THE DELIVERY AND PROTECTION EFFICIENCY OF RESPIRATORY MUCOSAL VACCINES

### 5.1 Adjuvants

Adjuvants are substances incorporated into vaccines to amplify their immunogenicity. Mucosal adjuvants can be classified into three main categories based on their functions: mucosal adhesion enhancers, permeation enhancers, and immune stimulators.^79^ Mucosal adhesion enhancers are compounds that can increase the retention time of pharmaceutical formulations on mucosal surfaces.^80^ These adjuvants improve the attachment and retention of vaccines on mucosal surfaces through physical and chemical interactions, thereby increasing vaccine stability and bioavailability. Common mucosal adhesion enhancers include chitosan, carboxymethylcellulose, hyaluronic acid, starch, sodium alginate, and hydrogel polymers. For example, Han Sang Yoo et al. demonstrated that chitosan nanoparticles carrying three recombinant Brucella proteins effectively promoted Th2‐type immune responses and the production of antigen‐specific IgA and various types of IgG in mouse models. This study underscores the role of chitosan nanoparticles in enhancing mucosal immunity and vaccine efficacy.^81^ Similarly, Fu et al.^57^ used a highly bioadhesive hydrogel as an adjuvant that confined the antigen to the surface of the nasal mucosa, thereby improving the stability of the antigen and prolonging its residence time in the nasal cavity and lungs.^57^ Permeation enhancers are a class of compounds that can increase the permeability of drugs or other substances through biological membranes, thereby improving their absorption efficiency.^82^ These agents facilitate the penetration of vaccines or other agents across biological barriers, enhancing antigen uptake and the immune response. Notable examples of permeation enhancers include cholera toxin and heat‐labile enterotoxin from Escherichia coli, which are well‐established in enhancing respiratory mucosal vaccine delivery.^83^ For example, Ye et al. used the pentameric cholera toxin B (CTB) subunit as a respiratory mucosal vaccine adjuvant and assembled it into CTB self‐assembled nanoparticles bearing the SARS‐CoV‐2 RBD. This approach supported alveolar delivery, continuous antigen release, and uptake by antigen‐presenting cells, thereby inducing specific IgG and IgA responses and local T‐cell responses.^84^ Yang et al. investigated the mucosal adjuvant activity of recombinant flagellin protein and its implications for nasal immunization strategies. Their results showed that the triple‐RBD scaffold protein with flagellin vaccine (3R‐NC) elicits enhanced efficacy of a multivalent recombinant SARS‐CoV‐2 RBD protein, resulting in robust mucosal immune responses and specific IgA responses in the upper respiratory tract.^85^, ^86^, ^87^ Immune stimulators, also known as immunostimulants or immunostimulators, are agents or compounds.^88^ These substances enhance the body's immune response to vaccine antigens through various mechanisms, such as improving the function of antigen‐presenting cells (APCs), activating the innate immune system, and promoting T‐cell‐ and B‐cell activity. Common immune stimulators include aluminum salts, Toll‐like receptors (TLRs) agonists, liposomes, and stimulators of interferon genes agonists. TLRs are critical pattern recognition receptors that are targeted by mucosal adjuvants. TLRs agonists primarily enhance antigen uptake by dendritic cells through TLRs binding, thereby initiating a range of immune responses, particularly Th1 responses.^79^ For example, the TLR3 agonist poly(I:C), a synthetic double‐stranded RNA analog, activates various APCs by mimicking viral RNA. Zheng et al. used poly(I:C) as an adjuvant to significantly boost both local and systemic immune responses upon nasal administration.^60^ STING play a critical role in the process of innate immune response by activating type I interferon signaling pathway. It is reported that STING agonists can serve as adjuvants of respiratory mucosal vaccines against influenza viruses, SARS‐CoV‐2, and streptococcus pneumoniae.^89^

In sum, the use of adjuvants in mucosal vaccines largely enhances their efficacy by improving both local and systemic immune responses, increasing antigen bioavailability, and reducing dose requirements and side effects, thereby providing more complete and durable protection.

### 5.2 Vectors

The delivery system of respiratory mucosal vaccines plays a critical role in transporting vaccine components to respiratory mucosal surfaces and associated immune tissues to elicit a potent and sustained immune response, overcoming the challenges posed by respiratory mucosal barriers while ensuring antigen stability and efficient delivery. Unlike intramuscular vaccines, respiratory mucosal vaccines require antigens to be presented in particulate form to be effectively recognized by APCs and to activate both local and systemic immune responses. Commonly used delivery vectors for respiratory mucosal vaccines include nanoparticles, liposomes, microneedle patches, virus‐like particles (VLPs), and virus vectors.^90^ Nanoparticles are tiny particles ranging in size from 1 to 100 nm. They are typically composed of biocompatible materials such as polylactic‐co‐glycolic acid, chitosan, or lipids.^91^ Nanoparticles can be engineered to modify their size and surface properties, affecting their distribution and uptake by the immune system. This customization enhances the stability and immunogenicity of the antigen. Recently, Wu et al. reported a ferritin nanoparticle vaccine (CePnF) displaying conserved SARS‐CoV‐2 antigenic epitopes. Intranasal immunization with CePnF in mice elicited strong humoral, cellular, and mucosal immune responses, along with durable immunity. In addition, antibodies induced by CePnF demonstrated cross‐reactivity and neutralizing activity against various coronaviruses including SARS‐CoV‐2 variants.^92^ Liposomes are tiny vesicles composed of lipid bilayers that can encapsulate both water‐soluble and lipid‐soluble antigens. This could improve antigen delivery by fusing with cell membranes or through endocytosis, thus prolonging the antigen's residence time on respiratory mucosal surfaces and increasing absorption efficiency.^93^ Baldeon et al. assessed an intranasal mRNA‐LNP encapsulated vaccine in Syrian hamsters, which induced IgG and IgA production, which significantly prevented lung pathology and body weight loss in Syrian hamsters.^94^ Microneedle patches are a novel drug delivery system consisting of an array of tiny needles, typically micrometers in size, that painlessly penetrate the outer layer of the skin.^95^ Consisting of arrays of microneedles made from metals, polymers, or carbohydrates, microneedle patches penetrate the epidermis to reach the dermis or submucosal layers. This method delivers antigens directly to areas rich in immune cells, enhancing antigen uptake and improving the overall immune response. VLPs and virus vectors are the most commonly used carriers in vaccine development, designed to mimic or directly induce viral infection within cells, making them highly recognizable by the immune system and capable of eliciting robust immune responses.^96^ Hassan et al. generated an adenoviral vector vaccine encoding the SARS‐CoV‐2 spike protein. When administered intranasally to K18‐hACE2 transgenic mice, this vaccine induced high levels of neutralizing antibodies, systemic and mucosal IgA and T‐cell responses and effectively prevented SARS‐CoV‐2 infection in both the upper and lower respiratory tract.^97^

In conclusion, these vectors offer unique advantages and are suitable for different application scenarios. By selecting the appropriate delivery system, the efficacy and protective capacity of respiratory mucosal vaccines can be significantly enhanced, ensuring that antigens and adjuvants effectively reach and remain at the respiratory mucosal surface to elicit the required immune response.

## 6 DISCUSSION

The significance of respiratory mucosal immunity, particularly in preventing viral infections, stems from its unique capability to target pathogens at their primary entry points‐respiratory mucosal surfaces.^98^ As the first line of defense, mucosal immunity provides a rapid and localized response to thwart pathogen invasion before viruses can establish systemic infections. Furthermore, respiratory mucosal immunity facilitates a specific and memory‐driven immune response against viral infections. The formation of memory B cells and T cells ensures that the immune system can respond swiftly upon re‐exposure to the same pathogen, providing long‐lasting protection.^99^ Additionally, effective mucosal immunity not only safeguards the individual but also reduces viral load and shedding, thereby lowering the risk of transmission to others. This is particularly critical during outbreaks of highly contagious viruses.

### 6.1 Benefits of an efficient combination of intramuscular and respiratory mucosal vaccines

The combination of respiratory mucosal and intramuscular vaccines is a strategy aimed at increasing the protective host immune response to combat viral infection by using multiple routes of immunization. Traditional intramuscular vaccines primarily induce pathogen‐specific antibodies through systemic humoral immunity. In contrast, novel respiratory mucosal vaccines focus on inducing mucosal immunity at local mucosal surfaces to combat viral invasion and prevent further spread. To address the issue of increasing immune escape capacity, respiratory mucosal vaccines complement intramuscular vaccines by providing additional protection against emerging variant strains of the respiratory viruses.

Acting as a heterologous booster for intramuscular vaccines, respiratory mucosal vaccines provide broad‐spectrum and longer‐lasting protection while stimulating the host–immune system through multiple mechanisms to combat various respiratory viruses. In particular, respiratory mucosal vaccines can induce robust local immune response at respiratory mucosal surfaces, such as the nasal cavity and respiratory tract, characterized by the production of secretory IgA. Furthermore, respiratory mucosal vaccines can also induce systemic humoral and cellular immune responses via mucosa‐associated lymphoid tissues and lymph nodes, providing multiple immunological protections that enhance the overall immune response. Additionally, respiratory mucosal vaccines can boost the activation and proliferation of several immune cells, particularly memory B and T cells, thereby enhancing long‐term immune memory.^100^ Given these benefits, combined inoculation regimens of intramuscular and respiratory mucosal vaccines utilize different mechanisms to enhance both local and systemic immune responses, thereby improving the efficacy of protection.

### 6.2 Changing the inoculation route from intramuscular to respiratory mucosal extends the efficacy and scope of approved vaccines

The development cycle for traditional intramuscular vaccines is typically long, often taking 10 to 15 years from research to market approval.^101^ However, in the event of an urgent pandemic outbreak, the vaccine development and approval processes can be accelerated. For example, for SARS‐CoV‐2 vaccines, emergency use authorization has significantly shortened development times, with some vaccines being developed and licensed within a year.^102^ Another important approach to accelerate the development and approval processes is altering the administration route of existing vaccines. The Ad5‐nCoV vaccine, originally licensed for intramuscular injection, has been adapted for nebulized inhalation in response to the continued emergence of SARS‐CoV‐2 variants. During the pandemic of SARS‐CoV‐2, this nebulized formulation was rapidly implemented in select regions and populations. Clinical studies have shown that nebulized Ad5‐nCoV induces robust local and systemic immune responses, producing IgA on respiratory mucosal surfaces and cellular immunity. In terms of safety, nebulized administration has shown a high safety profile, with adverse effects generally being mild and transient, such as mild irritation (e.g., cough or sore throat). Therefore, changing the route of inoculation is a promising strategy for accelerating vaccine development, particularly in the early stage of the pandemic.

### 6.3 Key technological breakthroughs and the potential of respiratory mucosal vaccines in combination with immunotherapies

With continuous advancements in medical science and technology, the field of vaccine development has been actively pursuing new innovations. Inhalable respiratory mucosal vaccines, as a novel vaccination method, have garnered significant attention. Compared with traditional intramuscular vaccines, respiratory mucosal vaccines provide a more convenient, safer, and easier‐to‐implement alternative, opening new possibilities for public health advancement.^103^ These innovations present a promising outlook for expanding the reach and impact of vaccination programs. However, the successful development of respiratory mucosal vaccines requires overcoming several significant technological hurdles. One of the primary challenges is the efficient delivery of antigens to respiratory mucosal surfaces. The mucosal environment is characterized by rapid clearance mechanisms, enzymatic degradation, and a thick mucus layer, all of which can impede antigen retention and immune activation.^104^ Thus, innovative vaccine delivery systems are essential to ensure antigen stability and promote efficient absorption. Additionally, a critical aspect of respiratory mucosal vaccine development is the need for potent adjuvants that specifically stimulate immune responses at mucosal surfaces.^105^ Currently, viral vector vaccines are the most widely used in respiratory mucosal vaccine research, leading to comparatively less emphasis on the development of adjuvants for these vaccines.^106^ The breakthroughs of adjuvant hold profound significance for respiratory mucosal vaccines. These advancements are essential for optimizing immune activation, enhancing the vaccine's protective efficacy, and ensuring long‐lasting immunity, thereby playing a pivotal role in the prevention of respiratory infections. Finally, Research techniques, such as single‐cell sequencing technology, provide powerful tools for the development of respiratory mucosal vaccines, enabling scientists to gain a more comprehensive understanding of immune responses and optimize them.^107^ Single‐cell technology allows for the precise analysis of individual immune cells within the respiratory mucosa, including T cells, B cells, and innate immune cells. This detailed examination offers valuable insights into the roles and interactions of these cells following vaccination. Such in‐depth analysis reveals the complex dynamics of immune responses, facilitating the optimization of vaccine design.^28^ By utilizing single‐cell RNA sequencing and mass spectrometry, researchers can identify critical cell subpopulations involved in effective immune responses. This enables the targeted development of more precise adjuvants and antigens, thereby improving vaccine efficacy. Furthermore, analyzing immune cell activation and cytokine secretion at the single‐cell level allows researchers to better understand and predict immune‐related side effects associated with vaccination, enhancing vaccine safety. In conclusion, single‐cell technology is transforming respiratory mucosal vaccine research by providing detailed insights into immune mechanisms and supporting the development of more effective and safer vaccines.

### 6.4 Potential adverse effects of respiratory mucosal vaccines

Respiratory mucosal vaccines offer several advantages over traditional intramuscular vaccines but also have potential adverse effects (Table 3). Localized adverse effects may include mild discomfort or irritation in the nasal cavity, throat, or airways, with severe cases possibly leading to bronchitis or asthma. Systemic reactions such as fever, tiredness, and headache may also occur. Furthermore, adverse reactions associated with certain types of respiratory mucosal vaccine carriers require careful consideration. For example, adenoviral vector vaccines have been associated with rare but serious side effects, such as thrombosis and thrombocytopenia.^108^ However, the safety of vaccine recipients and the efficacy of the vaccine can be maximized through well‐designed preventive measures, stringent surveillance mechanisms, and appropriate management strategies. Ongoing safety monitoring and feedback during vaccine administration will further optimize vaccination strategies.

**TABLE 3:** The advantages and disadvantages of respiratory mucosal immune vaccines.

Advantages	Potential disadvantages
Induction of mucosal immunity	Limited antigen exposure time
Noninvasive delivery	Shorter duration of immunity
Prevention of infection at the entry point	Potential for reduced efficacy in certain populations
Broad immune response	Risk of tolerance or weak immune response
Potential to reduce asymptomatic transmission	Complex regulatory and manufacturing processes

There are several key factors currently hindering its broader clinical application. One concern is the potential for intranasal vaccines to access the central nervous system through the olfactory nerve, which could result in adverse neurological effects.^109^ Although rare, this has been a concern with certain formulations, such as adjuvant intranasal influenza vaccines.^110^ To mitigate this risk, vaccines need to be carefully formulated to avoid components that could cross the blood–brain barrier. Another concern is the regulatory and approval hurdles, intranasal vaccines face stringent regulatory scrutiny, particularly due to concerns over safety and efficacy in large, diverse populations.^111^ The regulatory pathways for approval are often longer and more complex compared with traditional intramuscular vaccines, which can delay their availability for widespread use.

## 7 CONCLUSION AND FUTURE PROSPECT

Respiratory mucosal immunity is crucial for defense against respiratory virus infections. The development and use of respiratory mucosal vaccines offer several advantages, including enhanced local immunity, ease of administration, dual immune responses, and support for herd immunity, making them particularly effective in preventing respiratory and gastrointestinal infections. In addition to combating respiratory viral infections, respiratory mucosal vaccines hold promise for targeting other respiratory pathogens such as *Streptococcus pneumoniae* and *Klebsiella pneumoniae* aim to stimulate both local and systemic immune responses through nasal mucosal immunization, thereby enhancing resistance to infections caused by these bacteria. However, challenges such as antigen stability, pre‐existing immunity to virus vectors, local discomfort, and dose control remain to be addressed. Respiratory mucosal vaccines are noninvasive in nature, which reduces physical discomfort and improves vaccine acceptance. They also minimize the risks associated with needle reuse or accidents that could lead to blood‐borne infections and facilitate more frequent boosting. Furthermore, the absence of needles lowers costs and logistical requirements, potentially enabling self‐administration and supporting large‐scale immunization efforts during pandemics. With ongoing technological advances and a deeper understanding of immune mechanisms, respiratory mucosal vaccines are expected to play a more central role in public health strategies, providing broader and more effective protection.

## AUTHOR CONTRIBUTIONS

Ming Zhou and Haiqin Xiao contributed equally. Ming Zhou, Haiqin Xiao, and Xinyi Yang conceived and drafted the manuscript and generated the figures and tables. Xinyi Yang supported information organization and manuscript writing. Tong Cheng and Ningshao Xia edited and revised the manuscript. All authors have endorsed the final manuscript.

## CONFLICT OF INTEREST STATEMENT

The authors declare no conflict of interest.

## ETHICS STATEMENT

None.

## Data Availability

Not applicable.
